# Piperine attenuates cancer-associated pain induced by microglial activation via increasing miR-150-50p

**DOI:** 10.18632/aging.205908

**Published:** 2024-11-19

**Authors:** Yunlong Chen, Mianhua Wu

**Affiliations:** 1 Department of Oncology, Rudong County Hospital of Traditional Chinese Medicine, Rudong County 226400, Jiangsu, China; 2 Institute of Oncology, The First Clinical Medical College, Nanjing University of Chinese Medicine, Nanjing 210023, Jiangsu, China

**Keywords:** cancer pain, microglia, piperine, CXCL12, miR-150-5p

## Abstract

Aim: Severe painful neuropathy often occurs in cancer patients receiving chemotherapy. Emerging evidence has demonstrated that microglia contribute to the occurrence and development of cancer-associated pain. This study aimed to investigate the mechanisms by which piperine influences cancer-associated pain induced by microglia activation.

Methods: The tumor cell implantation (TCI) model was adopted as the cancer-associated pain model in mice. Behavioral tests were done to confirm that model mice were sensitive to acute mechanical and thermal pain. Western blot (WB) and immunofluorescence (IF) were conducted to quantify expression level of microglia marker protein Iba1 in mice spinal cord tissues. The expression of miR-150-5p and CXCL12 in the mice spinal cord was evaluated by Quantitative real-time Polymerase Chain Reaction (qRT-PCR) and fluorescence *in situ* hybridization (FISH). Primary microglia from mice were treated with lipopolysaccharide (LPS) to investigate neuroinflammation.

Results: The modeled mice showed high susceptibility to acute mechanical hyperalgesia and thermal hyperalgesia. The expression of microglia marker protein Iba1 in the model group was increased *in vitro* and *in vivo*. Treatment with piperine effectively relieved the cancer-associated pain in mice. The results of FISH and qRT-PCR showed that piperine significantly increased the expression of miR-150-5p and reduced the expression of CXCL12 in the spinal cord of mice. Furthermore, it inhibited the microglia-induced cancer-associated pain.

Conclusions: Piperine upregulates miR-150-50p levels, inhibits CXCL12 expression, and reduces microglia levels at the lesion site. Therefore, piperine may be a potential drug candidate for the treatment of cancer-associated pain.

## INTRODUCTION

The common types of cancers include breast, prostate, kidney, and lung cancer [1, 2]. Cancer tumors can easily metastasize to bones including the spine, femur, and tibia [3]. Tumor metastasis to bone leads to increased risk of infection, pain, hypercalcemia, anemia, bone fractures, spinal cord compression, and decreased mobility [4–6]. Nociception is one of the most common symptoms of patients with a malignant tumor. Therefore, the physical pain caused by malignant tumors affects the patient and their families mentally. This has severely affected the prognosis and quality of life of patients. Pain caused by cancer leads to suffering, anxiety, reduced confidence and general deterioration in patients with cancer [7]. It can be caused by tumor compression on nerves, side effects of certain cancer treatments such as surgery, chemotherapy and radiotherapy or a combination of factors [8]. Although the conventional “three-stage” drug cancer pain relief program proposed by the WHO has relieved the pain of many patients to a certain extent [9], there are several severe side effects associated with the drugs [10].

Several studies have reported on pain in cancer patients. However, the pathological mechanism of this type of pain is still unclear. It is evident that central nervous system (CNS) cells, such as microglia, astrocytes, and neurons, play an important role in the pathological process of pain [11, 12]. Microglia is a diverse population of macrophages that reside in the central nervous system. These macrophages critically influence tissue homeostasis [13]. Recently, several studies have reported on microglia in the field of pain research [14, 15].

During normal physiological conditions, microglia make use of its convoluted process to remove dead cells, protein aggregates, and other destructive residues in the CNS by phagocytosis and pruning off the redundant synapses. Therefore, microglia play a vital role in maintaining the health of the central nervous system [16, 17]. Microglia proliferation is the increase in the number of microglia in the spinal dorsal horn due to the microphages becoming hypertrophic after nerve injury. The reactive microglia release interleukins and other inflammatory factors. This stimulates immune cells and spinal cord neurons to respond accordingly and eventually leading to pathological pain signal transmission [18, 19].

In recent years, several studies have shown that cancer bone pain partly results from inflammation caused by the activation of microglia. After nerve injury, microglia can release a lot of pro-inflammatory mediators [20, 21]. These mediators activate the neuronal cognitive receptors and promote central sensitization, leading to spontaneous pain, hypoesthesia, and general anesthesia. In the case of peripheral neuropathic pain, these changes occur in spinal microglia and thus lead to cancer bone pain. Further, the microglia neuron interaction signal helps to inhibit the exciting pain-related pathway in the spinal cord, and hence promotes the progress of pain caused by cancer in the bones [22]. The involvement of microglia in neuropathic pain has been confirmed in rodent models. However, the response of microglia being the main pathological mechanism of cancer pain and whether it should be used as a drug target remains controversial [23, 24].

## MATERIALS AND METHODS

### Animals and experimental groups

The experimental animals used were C3H / HeN mice. The animals were males weighing between 15 and 30g. They were divided into two parts [25], one was pathological mechanism research, and the rest was for the efficacy evaluation of piperine. All the mice were acquired from SLAC Laboratory Animal (Beijing, China). The animals were housed in central animal facilities and maintained under controlled conditions of and kept at 22° C, light: dark (12:12 hours), and humidity (45 to 55%). Further, the mice were fed on a commercial diet and distilled water throughout the experimentation period. All the conducted animal procedures conformed to the NIH guide for the care and use of laboratory animals (NIH Publications No. 8023). The animal care and use committee of Nanjing University of Traditional Chinese Medicine provided the approval of the protocol used in this study (Registration No.: PZNJUCM202004556).

The 16 mice for pathological mechanism research were randomly subdivided into two groups (n=8), the sham group and Tumor cell implantation (TCI) group. The mice in the TCI group were treated with TCI surgical intervention whereas those in the sham operation group were only injected with an equal amount of cell culture medium. The pain caused by cancer was assessed at 0, 7, 14, and 21 days of the experiment period.

The 32 mice used for the pharmacodynamics mechanism of piperine were randomly divided into four groups (n=8), Sham, TCI, TCI + piperine, and piperine groups. The mice in TCI group and TCI + piperine group were treated with TCI surgical intervention. However, all the mice in the Sham operation group and piperine group were only injected with an equal amount of cell culture medium and then sacrificed on the 21st day after treatment.

### Cancer pain models after tumor cell implantation (TCI)

A total of NCTC 2472 mouse sarcoma cells (ATCC, USA) were cultured in NCTC-135 medium (Sigma Aldrich, USA) containing 10% fetal bovine serum (Cyclone, USA). The mice were anesthetized by intraperitoneal injection with pentobarbital sodium (50 mg/kg). An incision was made in the skin of the left patella after shaving and disinfecting the skin of the mice. The patellar ligament was cut off to expose distal femoral condyle. A 0.5 mm semicircular burr was used to drill into the medullary cavity. Nine needles and a 25 μL microinjector (sarcoma implanted mice) were used to inject the cell culture medium containing 1*10^5^ NCTC 2472 mouse sarcoma cells. Finally, the injection holes were sealed with bone wax, and the skin was sutured after disinfection as previously described [26, 27]. The mice in the sham group were injected with an equal volume of culture medium whereas the other operations were performed in the same manner as in the TCI group [28].

### Drug administration

There was no drug administration process in the pathological mechanism research study. On the other hand, piperine was dissolved in dimethyl sulfoxide (DMSO) and then diluted to 20 mg/mL solution with sterile saline for the study of pharmacokinetics mechanism of piperine. The final concentration of DMSO was 0.1% (v/v). The mice in the TCI + piperine and piperine groups were treated by daily gavage of piperine (80 mg/kg) at a fixed time for 21continuous days. The mice in the Sham and TCI groups were gavaged with solvent on a daily basis for 21 days [29].

### Pain behavior detection

Mechanical tenosynovitis was measured by the stimulated paw contraction threshold (PWT) response using von Frey’s (Stoelting, USA) [30]. The mice were put individually in cages and allowed to acclimate for 1 hour. The ascending von Frey hairs (0.4, 0.6, 1.4, 2, 4, 6, 8, and 15 g) were applied to the surface of the middle metatarsal of each hind paw. Each of them was kept for 5 to 6 seconds, with an interval of 5 minutes. A positive response was indicated by a brisk withdrawal or paw retraction upon stimulation. The experiment started with two grams of von Freis. Whenever there was a positive response to the stimulus, the next lower weight of von Freis was applied, and whenever there was a negative response, the next higher weight of von Freis is applied. The test included five more stimuli after the first change in response (positive or negative). Behavioral tests were blind treatment by a third-party researcher.

Thermal pain was determined by measuring paw withdrawal latency (PWL) in response to radiant thermal stimulation [31]. Plantar analgesia machine (IITC Life Science Inc., USA) was used to provide a heat source. Each mouse was enclosed in a plastic room with a transparent glass floor and allowed to acclimate to the environment for one hour before the start of experiments. The radiant heat source was transmitted through the glass floor of the plastic box and focused onto the hairless surface of the mouse’s paw. The machine was set to cut off automatically after 20 seconds of exposure to prevent tissue damage. Heat stimulation was delivered three times, on each hind paw at intervals of 5 minutes. The intensity of the thermal stimulus was maintained throughout the research process.

### Cell culture and treatment

Primary mouse microglia were purchased from ScienceCell (ScienceCell, USA) and cultured in a microglia culture medium (ScienceCell, USA) containing 5% fetal bovine serum. Mouse BV2 microbial cells were cultured in DMEM supplemented with 10% FBS containing 100 U/mL penicillin and 100 μg/mL streptomycin at 37° C in a 5% CO_2_ humidified incubator. The cells were treated with LPS (1 μg/mL) at the absence or presence of piperine (50 μM) for 0, 6, 12, 24, 48 h after the cells were seeded onto 6-well plates for 48 hours [32].

### Immunofluorescence

The mice were deeply anesthetized by subcutaneous injection of a mixture of ketamine and dextran. After thoracotomy, the heart was perfused using Cole Parmer Masterflex L/S peristaltic pump (model 7550-30). 4% paraformaldehyde and 0.12% picric acid were used for cold perfusion, after PBS perfusion. The lumbar spinal cord was removed at 4° C and fixed overnight. The tissue was dehydrated in 30% sucrose at 4° C, embedded in Tissue-Tek O.C.T. compound, and stored at – 80° C until further analysis was performed.

The spinal cord segments (L4-L5) of mice were continuously sectioned, at a thickness of 30 μm in a cryosectioner and loaded into a microscope slide. To remove impurities, the slices were washed in phosphate-buffered saline (PBS) for 5 minutes three times and in 0.1% Triton X-100 in PBS (T-PBS) for 5 minutes. For double immunostaining, sections were blocked in T-PBS with 5% donkey serum at room temperature for 1 hour and washed in T-PBS for 5 minutes three times. They were incubated with anti-iba1 (Cat: a19776) or anti-CXCL12 (Cat: a1325) antibody (dilution ratio: 1:500, ABclonal, China) at 4° C overnight. In the next day, the sections were washed in PBS for 5 minutes three times and incubated overnight with goat polyclonal antibody (1:1000, Cat: ab107159, Abcam, UK) at 4° C. On the third day, the slices were washed in PBS for five minutes three times, incubated with donkey anti-rabbit Alexa flow 594 (1:300, Cat: z25007, Thermo Fisher, USA) at room temperature for two hours, washed in PBS for five minutes four times and anti-stained with DAPI (1:300, Cat: d3571, Thermo Fisher, USA). After three washes in PBS for the last 5 minutes, the staining of Iba-1 or CXCL12 was observed under fluorescence microscope [33, 34].

### Fluorescence *in situ* hybridization

The L4-L5 spinal segments were fixed, paraffin-embedded, sliced, and baked into paraffin blocks. The paraffin blocks were dewaxed, and the antigen was repaired by adding protease K (20 μg/ml) to digest at 37° C for 10 minutes. After washing with pure water, PBS was washed for 5 minutes three times. The pre-hybridizing solution was added and incubated at 37° C for 1 hour. The pre-hybridization solution was poured out, and the hybridization solution containing probe was added and then kept hybridizing at 4° C overnight. After washed at 37° C for 10 minutes, the hybridizing solution was washed away, and mice anti digoxin labeled 488 was added. The antibody was added after the blocking solution was removed and then incubated at 37° C for 50 minutes. Phosphate buffered saline was washed for 5 minutes four times and DAPI staining solution was added to the slices and incubated in dark for 8 minutes. After washing, an anti-fluorescence quenching blocking agent was added to seal the slices. The sections were observed, and images collected under Nikon fluorescence microscope (UV excitation wavelength 330-380 nm, emission wavelength 420 nm, blue light); Fam (488) emits green light with an excitation wavelength of 465-495 nm and emission wavelength of 515-555 nm; excitation wavelength of Cy3 red light is 510-560 nm, and emission wavelength is 590 nm [35].

### Dual-luciferase experiments

To construct a luciferase reporter vector, CXCL12 cDNA was amplified using PCRʹ-UTR, which contains the predicted potential mir-150-5 p binding site, was sub-cloned downstream of the luciferase gene in pmirGLO dual luciferase vector (Promega, USA). Primary mouse microglia were cultured in 24 well plates and co-transfected with 50 ng firefly luciferase containing vector and 25 ng mir-150-5 p mimic or control. Transfection was performed using Lipofectamine 2000 reagent (Invitrogen, Germany). At 48 hours after transfection, the fluorescence of firefly and Renilla were normalized using dual luciferase reporter (Promega, Fitchburg, WI, USA) [36].

### Quantitative real-time polymerase chain reaction (qRT-PCR)

Quantitative real-time polymerase chain reaction was used to analyze the expression of miRNA and mRNA in the mouse spinal cord. Total RNA was extracted using Trizol reagent (Takara, China). The reverse transcription reaction was performed using a frequent RT Kit (Tiangen, China). The primers were designed and synthesized by Shanghai biotechnology. Quantitative real-time polymerase chain reaction was performed according to the instructions of the PCR kit (Tiangen, China). U6 and GAPDH were used as internal references of miRNA and mRNA, respectively. The relative quantitative method (2^- ΔΔ CT^ method) [37].

### Western blotting (WB)

The L4-L5 spinal segments were immediately removed, homogenized in cold protein lysate, and centrifuged (12000 rpm) for 30 minutes at 4° C. To determine the protein concentration, BCA protein detection kit (Thermo Scientific, USA) was used. The protein was boiled in a loading buffer at 90° C for 8 minutes and stored at - 80° C. Protein samples (30 to 50μg) were added and separated on 10% twelve alkyl sulfate-polyacrylamide gel electrophoresis and then transferred to the PVDF membrane. The membrane was sealed with 5% bovine serum albumin in Tris-buffered saline and Tween 20 (TBST, 0.1%) at room temperature for 2 hours, and then incubated with the specific main antibody at 4° C overnight, including anti-iba1 (Cat: a19776), anti- β- Actin (Cat: ac026) and anti-CXCL12 (Cat: a1325) (dilution ratio 1:1000, ABclonal, China). After washing in TBST, Goat anti-mouse second antibody (1:5000, ABclonal, China) combined with horseradish peroxidase (HRP) was incubated at room temperature for 2 hours. Finally, SuperLumia ECL Plus HRP Kit (K22030, Abbkine, China) was used to detect the protein bands by Bio-Rad ChemiDoc XRS and chemiluminescence imaging system (Bio-Rad, USA). A system with image lab software (Bio-Rad Laboratories, USA) was used to analyze the band strength [38].

### Statistical analyses

All statistical analyses were performed using SPSS 17. The data from these experiments were presented as Mean ± Standard Error of Mean (SEM). The data from different groups was also subjected to inferential statistics using one-way ANOVA. The statistically significant difference was reported at *P*<0.05.

### Availability of data and materials

The data generated or analyzed during this study are included in this article, or if absent are available from the corresponding author upon reasonable request.

### Consent for publication

Consent for publication has been obtained from all authors.

## RESULTS

### Effects of cancer pains on microglia in the spinal cord of mice

**Figure 1:**
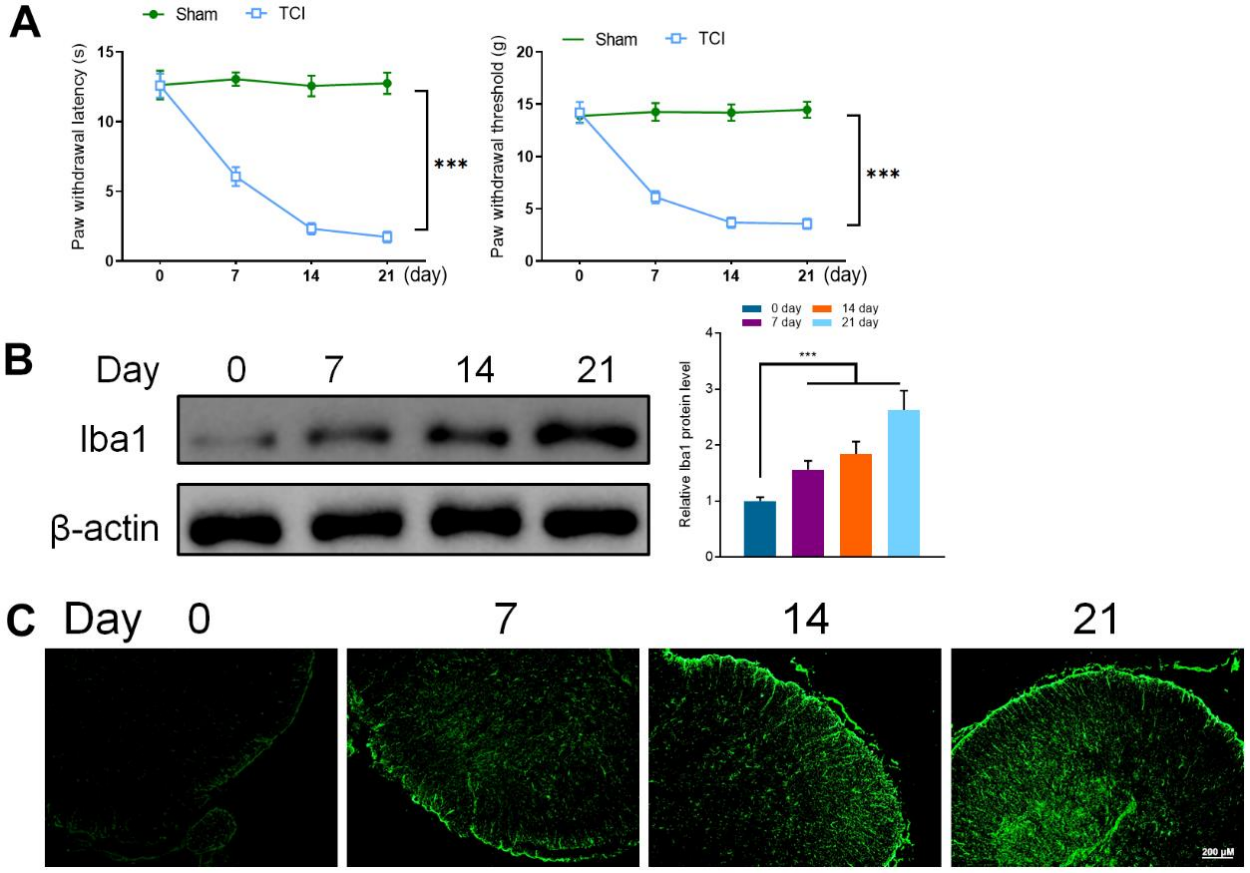
(**A**) Hind paw withdrawal reflex latency and threshold. (**B**) Western Blot detected the expression of Iba1 (0, 7, 14, and 21 days). (**C**) Immunofluorescence was utilized to detect the expression of Iba1 (0, 7, 14, and 21 days). Values were expressed as means ± SEM, n=8, *** *P*<0.001.

To verify the successful induction of TCI model mice, PWT and PWL were used to determine the mechanical and thermal response of mice. Although the PWT and PWL of the mice in the TCI group started to decrease at the 7th day after induction, the change was insignificant in the sham group. These results indicated that the cancer pain model was successful (Figure 1A).

To verify the role of microglia in the spinal cord of mice with the cancer pain model, the spinal cord samples of mice were taken on 0, 7, 14, and 21 days after induction. Western blotting and immunofluorescence were used to detect the expression of Iba1, a marker of microglia activation in the spinal cord of mice. It was found that the expression of Iba1 in the spinal cord of mice increased significantly on the 7th day and continued to increase on the 14th and 21st days of the experiment period. These results confirmed that cancer pain can lead to the activation of microglia in the spinal cord (Figure 1B, 1C).

### Effects of microglia activation on miR-150-5p expression in the spinal cord of mice

**Figure 2:**
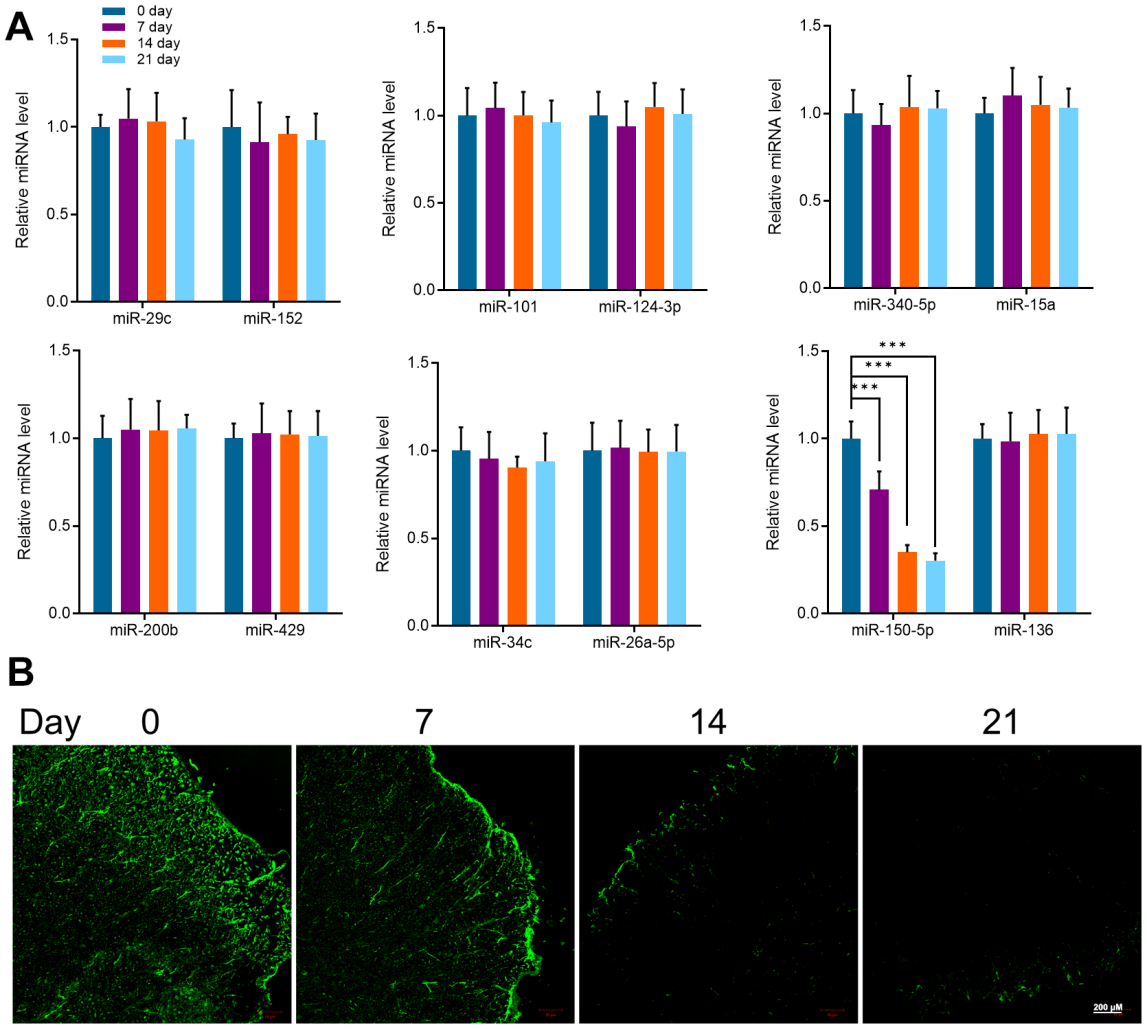
(**A**) qRT-PCR detected the expression of miR-29c, miR-152, miR-101, miR-124-3p, miR-340-5p, miR-15a, miR-200b, miR-429, miR-34c, miR-26a-5p, miR-150- 5p and miR-136 in mice spinal cord at different periods (0, 7, 14, and 21 days). (**B**) FISH detected the expression of miR-150-5p in the spinal cord of mice at different periods (0, 7, 14, and 21 days). Values were expressed as means ± SEM, n=8, *** *P*<0.001.

It has been reported that miRNAs are considered as “gene switches” of microglia phenotype in the spinal cord. Most miRNAs directly target their mRNA, downregulate the protein expression of microglia, and prevent the progress of pain. In recent years, due to the progress of sequencing technology, miRNA expression profiles of microglia in different types of pain have been found and confirmed. Therefore, in this study, miR-29c, miR-152, miR-101, miR-124-3p, miR-340-5p, miR-15a, miR-200b, miR-429, miR-34c, miR-26a-5p, miR-150- 5p and miR-136 were detected by qRT-PCR in the spinal cord of mice at different stages [39–43]. Interestingly, the expression of miR-150-5p gradually decreased with time while the other miRNA did not change remarkably. Compared with day 0, the expression of miR-150-5p was significantly decreased at 7, 14, and 21 days (Figure 2A). Further, the expression of miR-150-5p detected by fluorescence *in situ* hybridization (FISH) was consistent with the result of qRT-PCR (Figure 2B). Compared with Iba1, the expression of miR-150-5p was significantly decreased after cancer pain and there was a negative correlation between the two. It is suggested that the expression of mir-150-5p is related to microglial activation induced by the pain caused by cancer.

### Effects of overexpression of CXCL12 on microglia

**Figure 3:**
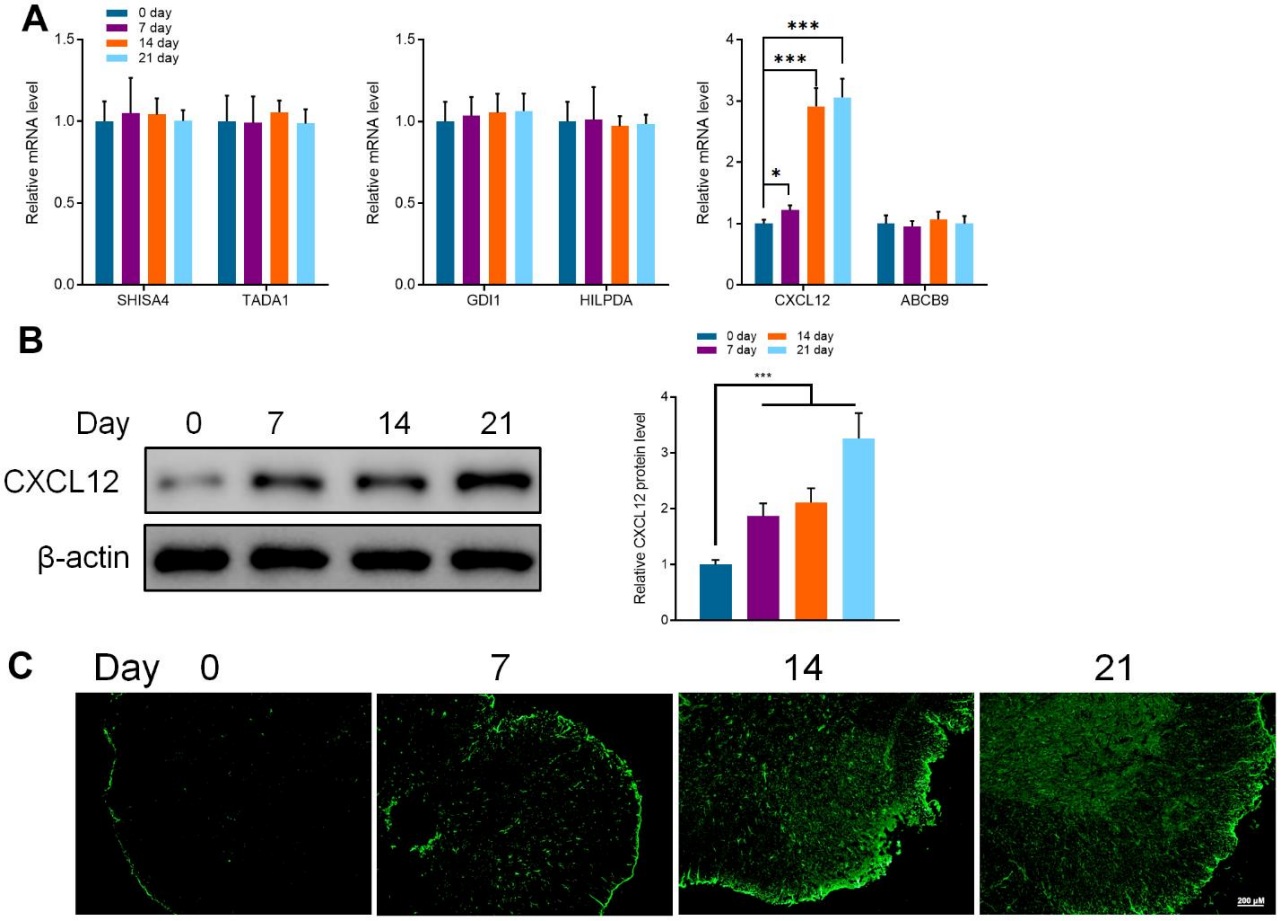
(**A**) qRT-PCR detected the mRNA expression of SHISA4, TADA1, GDI1, HILPDA, CXCL12, and ABCB9 in the spinal cord of mice at different periods (0, 7, 14, and 21 days). (**B**) Western Blot detected the protein expression of CXCL12 in the spinal cord of mice at different periods. (**C**) Immunofluorescence was used to detect the protein expression of CXCL12 in the spinal cord of mice at different periods (0, 7, 14, and 21 days). Values were expressed as means ± SEM, n=8, * *P*<0.05, *** *P*<0.001.

The databases including miRanda [44] (http://regendbase.org/tools/miranda), targetScan [45] (https://www.targetscan.org/vert_71) and RNA-hybrid (https://alk.ibms.sinica.edu.tw/cgi-bin/RNAhybrid/RNAhybrid.cgi) were used to analyze and predict the downstream target genes of miR-150-5p, Shisa family member 4 (SHISA4), transcriptional adaptor 1 (TADA1), GDP dissociation inhibitor 1 (GDI1), hypoxia-inducible lipid droplet-associated (HILPDA), Chemokine CXC motif ligand 12 (CXCL12) and ATP-binding cassette, sub-family B (MDR/TAP), member 9 (ABCB9) were its potential target genes. Therefore, we utilized qRT-PCR to detect the mRNA expression of these genes. It was noted that the expression of CXCL12 was decreased while the other mRNA did not change significantly. Compared with day 0, the expression at the 7, 14, and 21days were significantly increased (Figure 3A). Further, the protein expression of CXCL12 was detected by WB and IF, and qRT-PCR. Expression of CXCL12 was sharply increased after the occurrence of cancer pain, and it was positively correlated with the expression of Iba1. This implies that the expression of CXCL12 was related to the activation of microglia caused by cancer pain (Figure 3B, 3C).

### Inhibition effects of piperine on microglia

**Figure 4:**
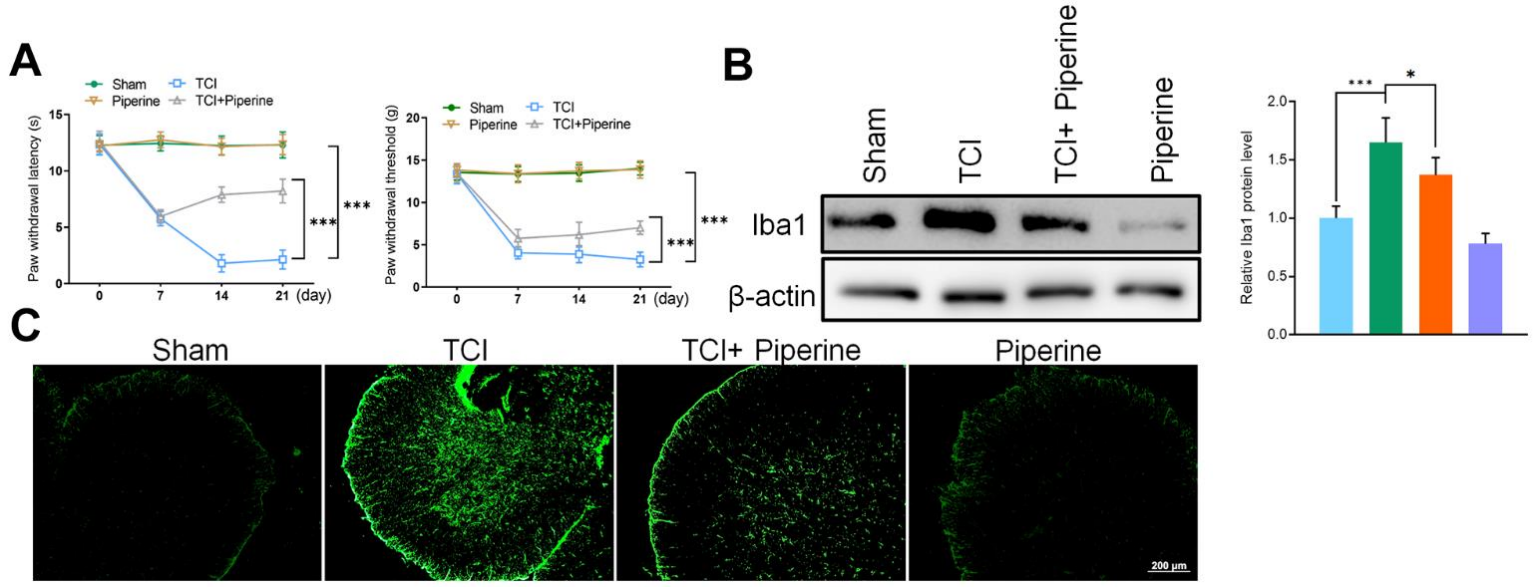
(**A**) Hind paw withdrawal reflex latency and threshold. (**B**) Western Blot detected the expression of the marker protein Iba1 in the spinal cord of mice. (**C**) Immunofluorescence detected the expression of the marker protein Iba1 in the spinal cord of mice. Values were expressed as means ± SEM, n=8, *** *P*<0.001.

According to the results of mice behavior, the PWL and PWT of mice in the TCI group decreased gradually after 7, 14 and 21 days. However, piperine caused significant reversal of these changes (*P*<0.001), and with the prolongation of time. Piperine could have enhanced the mechanical sensitivity and thermal sensitivity of mice in the TCI group and had a certain analgesic effect on cancer pain in the mice. In addition, piperine alone did not affect the behavioral test results of mice in the sham operation group, and this indicated that piperine was safe and did not affect the mice (Figure 4A).

To investigate the effect of piperine on the activation of spinal microglia, the spinal cord of all mice was taken out to test for the expression of Iba1 (including sham group, TCI group, TCI + piperine group and piperine group) on day 21. Results of this study revealed that the expression of Iba1 in the spinal cord of mice in the TCI group was significantly activated, which also confirm the activation of microglia in spinal cord. The results of western blot and IF also revealed that the expression of Iba1 was significantly decreased after piperine treatment. Therefore, this was an indication that piperine inhibited the activation of spinal microglia in mice with cancer pain (Figure 4B, 4C).

### Effects of piperine on the expression of miR-150-50p and CXCL12 in spinal cord of mice

**Figure 5:**
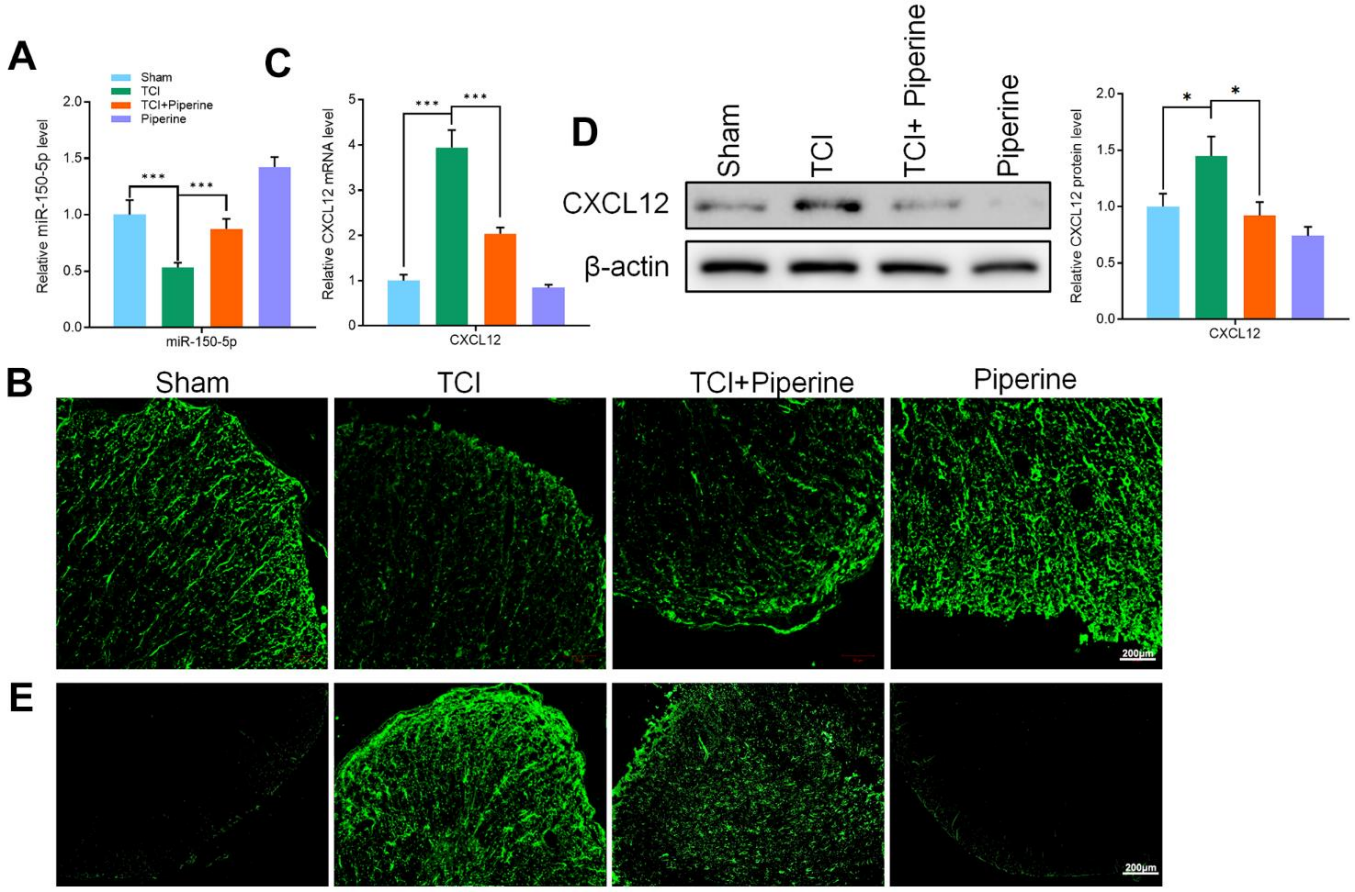
(**A**) qRT-PCR detected the expression of miR-150-50p in the spinal cord of all mice. (**B**) FISH detected the miR-150-50p expression in the spinal cord of mice. (**C**) qRT-PCR detected the CXCL12 expression in the spinal cord of mice. (**D**) Western blot detected CXCL12 protein expression in the spinal cord of mice. (**E**) Immunofluorescence detected CXCL12 protein expression in the spinal cord of mice. Values were expressed as means ± SEM, n=8, ** *P*<0.01, *** *P*<0.001.

The qRT-PCR and FISH technologies were used to detect the expression of miR-150-50p in the spinal cord of mice in all groups. It was found that the expression of miR-150-50p in the spinal cord of mice in the TCI group was significantly decreased. However, the levels of miR-150-50p were increased in the spinal cord of mice after treatment with piperine (Figure 5A, 5B). Piperine reversed the increase in CXCL12 protein (Figure 5C, 5E) and mRNA (Figure 5D) expression in mice. It was noted that piperine can significantly inhibit the expression of CXCL12 in the spinal cord. Further, the results of this study showed that piperine can inhibit the expression of CXCL12 by promoting the expression of mir-150-5p in the spinal cord of mice. Therefore, the TCI induced activation of spinal microglia was inhibited, and the pain caused by cancer was hence improved.

### Effects of lipopolysaccharide on the expression of miR-150-50p and CXCL12 in microglia

**Figure 6:**
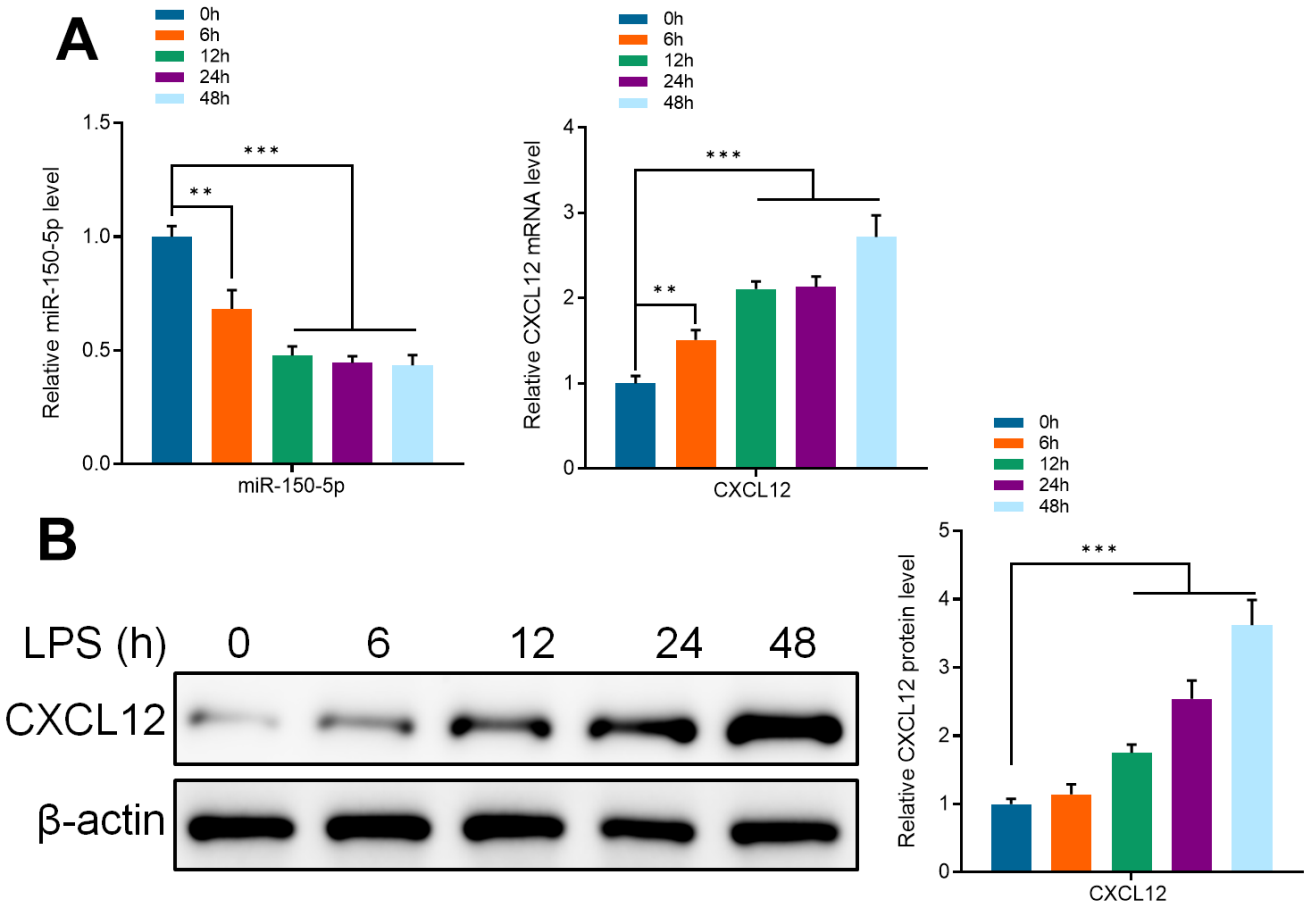
(**A**) qRT-PCR detected the expression of miR-150-5p and CXCL12 in microglia after incubating in LPS (1 μg/ml) for different periods (0, 6, 12, 24 and 48 hours). (**B**) Western Blot detected the protein expression of CXCL12 in microglia after incubating in LPS (1 μg/ml) for different periods. Values were expressed as means ± SEM, n=3, ** *P*<0.01, *** *P*<0.001.

Several studies have reported that lipopolysaccharide (LPS) can activate microglia *in vitro*. Therefore, to confirm the mechanism of piperine on the activation of microglia, primary mice microglia were treated with LPS (1μg/ml) and the detection time points were set as 0, 6, 12, 24, and 48 hours. Through qRT-PCR detection, it was found that the expression of miR-150-5p was significantly decreased at 6 hours after LPS stimulation. The decrease in expression of miR-150-5p was continued at 12, 24, and 48 hours. These results indicated that miR-150-5p played an important role in the activation of microglia in mice (Figure 6A). That the mRNA and protein expression of CXCL12 was significantly increased after LPS activating at every observation period, shows that CXCL12 was directly involved in the activation of microglia in mice (Figure 6B).

### Effects of piperine on the expression of miR-150-50p, CXCL12 *in vitro* in microglia

**Figure 7:**
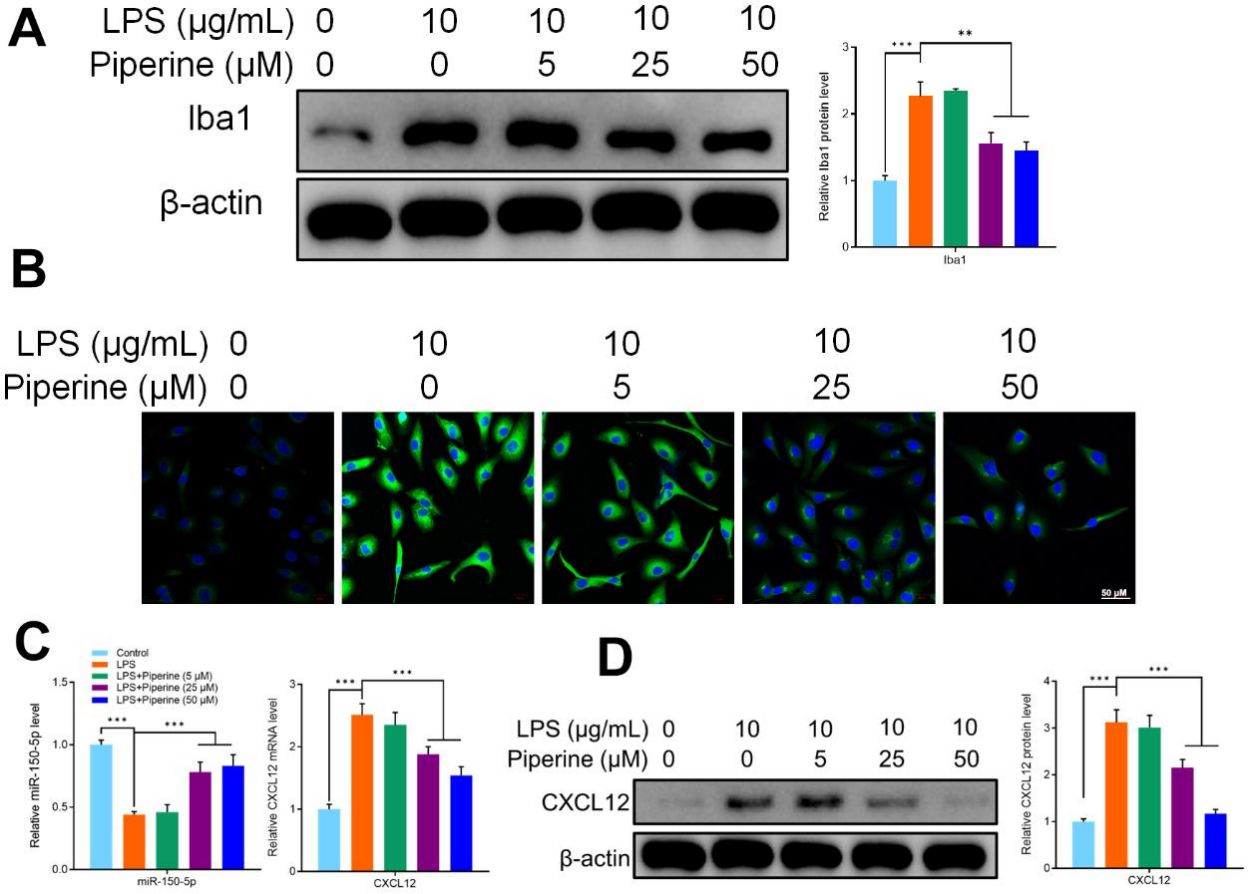
(**A**) Western blot detected Iba1 expression of different groups of mouse primary microglia. (**B**) Immunofluorescence detected Iba1 expression of different groups of mouse primary microglia. (**C**) qRT-PCR detected the expression of miR-150-5p and CXCL12 in different groups of mouse primary microglia. (**D**) Western blot detected CXCL12 expression of different groups of mouse primary microglia. Values were expressed as means ± SEM, n=3, ** *P* < 0.01, *** *P* < 0.001.

To determine the effect of piperine on the activation of microglia *in vitro*, primary microglia of mice were treated with 5, 25 or 50 μM piperine for 15 minutes, and then LPS (1 μg/ml) was used to stimulate the cells for 24 hours. Immunofluorescence and western blot were used to detect the expression of Iba1 in primary microglia of mice in all groups. The results of this study showed that LPS activated the expression of Iba1 in primary microglia of mice. The expression of Iba1 decreased significantly after piperine treatment (Figure 7A, 7B). This indicates that piperine inhibited the activation of primary microglia in mice. Experiments in this study had shown that piperine could promote the expression of miR-150-5p and inhibit the expression of CXCL12 in the spinal cord of in mice. The expression of miR-150-50p and CXCL12 was detected by qRT-PCR. It was found that LPS inhibited the expression of miR-150-50p while the expression of miR-150-50p increased after piperine treatment. The results of qRT-PCR showed that LPS activated CXCL12 mRNA expression in primary microglia of mice. Similarly, WB results showed that LPS activated the protein expression of CXCL12 in primary microglia of mice. As we expected, piperine could reverse these results in mouse primary microglia (Figure 7C, 7D).

### Feedback regulation effects of MiR-150-50p (in microglia) on CXCL12

**Figure 8:**
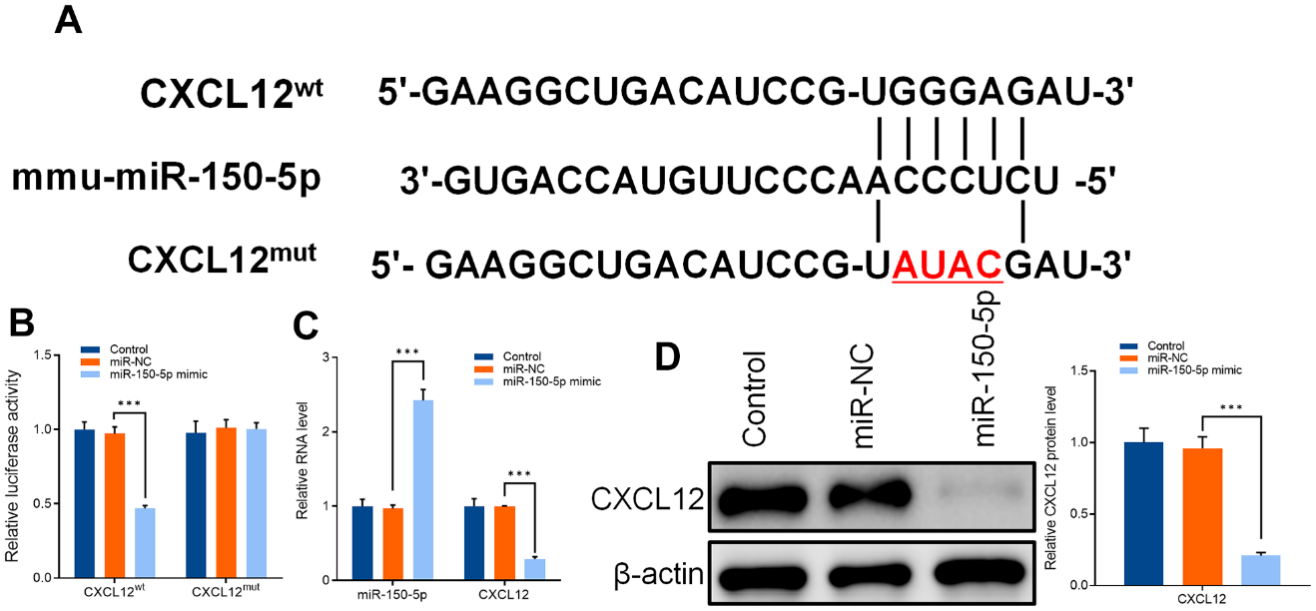
(**A**) Sequences of mmu-miR-150-5p and CXCL12-wild type (CXCL12^WT^) and CXCL12-mutant type (CXCL12^MUT^). (**B**) Dual luciferase assay was used to detect the activity of CXCL12 wild type (CXCL^WT^), CXCL12 mutant (CXCL^MUT^), and mir-150-5p overexpression vector (mir-150-5p mimic) after transfection into primary microglia of mice. (**C**) qRT-PCR was used to detect the expression of mir-150-5p and CXCL12 in mouse primary microglia after transfection of miR-NC and miR-150-5p. (**D**) Western blot was used to detect the protein expression of CXCL12 in primary microglia of mice after transfection of miR-NC and miR-150-5p. Values were expressed as means ± SEM, n=3, *** *P* < 0.001.

To verify the mechanism in the relationship between mir-150-5p and CXCL12an additional research was carried out. The potential binding sites of miR-150-5p and CXCL12 were analyzed by database (https://www.targetscan.org/vert_71) (Figure 8A). The overexpression of miR-150-5p decreased the fluorescence intensity of primary microglia of mice transfected with CXCL12 wild-type vector. However, CXCL12 mutant vector had no effect on the fluorescence intensity of primary microglia of mice (Figure 8B). Western blot and qRT-PCR results also confirmed that CXCL12 protein and mRNA levels were inhibited after miR-150-5p overexpression in primary microglia of mice (Figure 8C, 8D).

## DISCUSSION

In recent years, blocking of the abnormal activation of microglia has been reported by many researchers for the treatment of nociception conditions [46, 47]. With the development of new research technology, the clinical application of microglia-targeted therapy has potential hope for patients with pain caused by cancer [48]. Because of the natural characteristics of the chemical structure of miRNAs, they provide a possible method for the treatment of various diseases. They may also become a new treatment strategy for various types of pain, especially cancer pain [49]. They are often used as anti-inflammatory regulators to change the phenotype of microglia and macrophages [50].

Activation of microglia by M1 macrophages can aggravate the injury and gradually destroy the tissue. On the other hand, activation of microglia by M2 macrophages can inhibit inflammation and promote tissue repair. Therefore, regulation of the balance between pro-inflammatory (M1-like phenotype) and anti-inflammatory (M2-like phenotype) should be as much as possible toward neural repair response [51]. Currently, the biggest challenge for miRNA therapy is in its small volume [52]. It has several specific downstream targets. Therefore, this limits the application of miRNAs in the treatment of cancer pain, by interfering with other physiological functions and producing side effects [53].

The results of this study showed that piperine could activate the expression of miR-150-5p in the mouse spinal cord. However, the pharmacological mechanism of piperine is still unclear [54, 55]. Future research should be conducted to determine the precise interaction among piperine, protein, and miRNA and clarify its mechanism of action [56, 57]. CXCL12/CXCR4 chemokine signals play a key role in regulating diverse nervous system development processes and synaptic plasticity. The signal imbalance may be the root cause of central nervous system dysplasia, carcinogenic process, and stroke [58]. Recently, this chemokine signal has attracted much attention for its new involvement in pain signal regulation. Previous studies have shown that inoculation or intrathecal injection of exogenous CXCL12 into rats can promote continuous mechanical pain [59]. In similar pain-related models, the expression of CXCL12 or CXCR4 was significantly increased in DRG or spinal cord [60]. Blocking CXCR4 can reverse pain-related behaviors. In addition, CXCL12/CXCR4 signaling has been confirmed to mediate the release of TNF-α, NF-κB, and IL-6 in glial cells, which leads to additional pain sensitization [61]. However, whether CXCL12/CXCR4 signals contribute to pain hypersensitivity in the state of bone cancer is still largely unexplored. In addition, neuronal sensitization associated with glial activation in the spinal cord is also important for the development and maintenance of pain hypersensitivity in the state of bone cancer. Several studies have evidently shown that chemokines (such as CCL2, CCL5, CXCL1, and CX3CL1) are involved in neuronal sensitization or microglia activation in the spinal cord, and some of these chemokines contribute to the development of cancer pain [62, 63]. The results of this study also found that the mRNA and protein expression of CXCL12 was remarkably increased in the spinal cord of mice with pain caused by tumor cell implantation. Further, the trend is consistent with the expression of the microglia, and its expression is markedly reduced after the intervention of piperine. Therefore, we speculate that targeted inhibition of CXCL12 may alleviate cancer-associated pain.

Prostaglandins, tumor necrosis factors, and other factors can increase the sensitivity of tumor tissues or dorsal root ganglion [64]. Persistent stimulation of tumor tissues may transform sensory structure of the spinal cord and brain to be high-level center sensitive. The CXCL12/CXCR4 axis plays an important role in various aspects of cancer biology, including angiogenesis and metastasis. The role of CXCL12/CXCR4 axis in pain management after tumorigenesis has been investigated. In animal models of cancer pain, the CXCL12 / CXCR4 axis has been found to mediate the effects of astrocytes on cancer-associated pain. Since CXCL12 is mainly expressed in spinal cord astrocytes after tumor cell implantation, intrathecal injection of astrocyte inhibitor or selective JNK inhibitor can block the tumor cell implantation induced by high expression of CXCL12 in the spinal cord [65, 66]. Other studies found that intrathecal injection of CXCL12 neutralizing antibody alleviated cancer pain and reduced Iba1 levels after TCI treatment. The results indicate that the microglia may be involved in the treatment of central nervous system cancer pain mediated by CXCL12/CXCR4. In addition, it was found that the TCI model had higher levels of phosphorylated MAPK in the lumbar spinal cord and pro-inflammatory cytokines (IL-1β, IL-6, and TNF-α) [67]. Thus, blocking CXCL12 or MAPK may prevent the effects of CXCL12 / CXCR4 axis hence reversing the persistent pain associated with cancer. The present results suggest that the MAPK signaling pathway in the spinal cord may influence the CXCL12 / CXCR4-mediated neuroinflammation in tumors. Given its anti-inflammatory effect, we speculate that piperine may inhibit neuroinflammation thereby improving cancer-associated pain.

## CONCLUSIONS

This study shows that Piperine treatment decreases cancer-associated pain induced by tumor cell implantation. Importantly, piperine did not trigger any side effects on the behavioral function of normal mice. Mechanistically, piperine upregulated miR-150-5p levels in the spinal cord and decreased microglia activation by inhibiting CXCL12.
